# Predictive value of miRNA-21 on coronary restenosis after percutaneous coronary intervention in patients with coronary heart disease: A protocol for systematic review and meta-analysis

**DOI:** 10.1097/MD.0000000000024966

**Published:** 2021-03-12

**Authors:** Haiyue Dai, Jun Wang, Zhongping Shi, Xiaojun Ji, Yiwei Huang, Rui Zhou

**Affiliations:** a Wenzhou Central Hospital; b Third Clinical Institute Affiliated to Wenzhou Medical University, Wenzhou People's Hospital, Wenzhou, Zhejiang province, China.

**Keywords:** biomarker, coronary heart disease, coronary restenosis, diagnosis, meta-analysis, miRNA-21, percutaneous coronary intervention

## Abstract

**Background:**

Evidence reveals that microRNA (miRNA) can predict coronary restenosis in patients suffering from coronary heart disease (CHD) after percutaneous coronary intervention (PCI). Perhaps, miRNA-21 is a promising biomarker for the diagnosis of coronary restenosis after PCI. However, the accuracy of miRNA-21 has not been systematically evaluated. Therefore, it is necessary to perform meta-analysis to certify the diagnostic values of miRNA-21 on coronary restenosis after PCI.

**Methods:**

China National Knowledge Infrastructure, Wanfang, VIP, and China Biology Medicine disc, PubMed, EMBASE, Cochrane Library, and Web of Science were searched for relevant studies to explore the potential diagnostic values of miRNA-21 on coronary restenosis after PCI from inception to January 2021. All data were extracted by 2 experienced researchers independently. The risk of bias about the meta-analysis was confirmed by the Quality Assessment of Diagnostic Accuracy Studies-2. The data extracted were synthesized and heterogeneity was investigated as well. All of the above statistical analyses were carried out with Stata 16.0.

**Results:**

This study proved the pooled diagnostic performance of miRNA-21 on coronary restenosis after PCI.

**Conclusion:**

This study clarified confusions about the specificity and sensitivity of miRNA-21 on coronary restenosis after PCI, thus further guiding their promotion and application.

**Ethics and dissemination:**

Ethical approval is not required for this study. The systematic review will be published in a peer-reviewed journal, presented at conferences, and shared on social media platforms. This review would be disseminated in a peer-reviewed journal or conference presentations.

**OSF Registration Number:**

DOI 10.17605/OSF.IO/356QK.

## 1 Introduction

According to the statistics of the World Health Organization (WHO), more than 17.5 million people died of cardiovascular disease in 2015, accounting for approximately 31% of the global mortality rate.^[1]^ This number is expected to reach to 23 million by 2030.^[1]^ Coronary heart disease (CHD) is a cardiovascular disease caused by a variety of factors, and is also one of the common causes of death in the world.^[2–5]^ At present, its pathogenesis is not completely clear. Percutaneous coronary intervention (PCI) is an effective technique for the clinical treatment of CHD, and can greatly improve the survival rate of patients with CHD.^[6,7]^ However, the problem of in-stent restenosis seriously influences the effects of PCI, which still perplexes clinical workers.^[8]^ Related studies have displayed that 20% of patients after PCI have adverse ischemic events such as in-stent restenosis or thrombosis.^[9]^ Coronary angiography is still the gold standard for the evaluation of restenosis, with invasiveness and some complications. Therefore, it is of significantly clinical significance to find biochemical markers that can predict the prognosis of patients with stent implantation and even diagnose restenosis.

MicroRNA (miRNA) is a short non-coding RNA fragment that binds to messenger RNA^[10,11]^ and it plays a different role in many physiological or pathological process. Encoded by MIR21 gene, MiRNA-21 is one of the earliest discovered miRNA and located on the chromosome positive chain 17q23.2.^[12]^ MicroRNA-21 is transcribed by polymerase II and has its own promoter sequence, and exists in cells and out of cells. Meanwhile, it can regulate many signal transduction pathways and involves in endothelial cell differentiation, migration and angiogenesis.^[13–16]^ Therefore, it is significant in cardiovascular system. It can be interesting to apply it as a biomarker, especially for cardiovascular disease.

MiRNA-21 is important in many biological processes, including cardiovascular system. Same as all miRNAs, miR-21 is regulated at the posttranscriptional level by various regulatory proteins, including TGF-β receptor, phosphatase and tensin homolog, and Smad7.^[17]^ MiRNA-21 refers to one of the microRNA and is highly expressed in endothelial cells, so it is involved in endothelial cell differentiation, migration and angiogenesis.^[18]^ Therefore, miRNA-21 may be a potential auxiliary biomarker for the diagnosis and prognosis of cardiovascular diseases.^[19]^

Previous studies have confirmed the abnormal expression of miRNA-21 in patients with coronary restenosis after PCI, indicating that miRNA-21 has potential diagnostic value.^[20,21]^ Current evidence suggests that miRNA-21 may be a new biomarker and potential therapeutic target for the prediction of coronary restenosis in patients with CHD after PCI, but there are different reports.^[21–24]^ Therefore, this study adopts the method of meta-analysis for quantitative and comprehensive statistics to comprehensively and objectively evaluate the predictive value of miRNA-21 in patients with CHD after PCI.

## 2 Methods

### 2.1 Study registration

The protocol of the systematic review has been registered on Open Science Framework (registration number: DOI 10.17605/OSF.IO/356QK). It was reported by following the guideline of Preferred Reporting Items for Systematic Reviews and Meta-analysis Protocol statement.^[25]^

### 2.2 Inclusion criteria for study selection

#### 2.2.1 Type of studies

To explore the diagnostic value of miRNA-21 on the diagnosis of coronary restenosis after PCI.

#### 2.2.2 Type of participants

All patients with CHD after PCI were included.

#### 2.2.3 Type of index test

Index test: miRNA-21 was applied to detect patients with coronary restenosis after PCI. However, we excluded case reports, reviews, cell, or animal studies.

#### 2.2.4 Outcome measurements

Outcomes include pooled sensitivity (SEN), specificity (SPE), positive likelihood ratio, negative likelihood ratio, diagnostic odds ratio, area under the curve, and their 95% confidence intervals (CIs).

### 2.3 Data sources and search strategy

This study conducted a literature search in China National Knowledge Infrastructure, Wanfang, VIP, and China Biology Medicine disc, PubMed, EMBASE, Cochrane Library, and Web of Science. We made a final search on January 2021. The search strategy of Pubmed is displayed in Table 1.

**Table 1:** PubMed search strategy.

Number	Search terms
#1	Coronary Disease [MeSH]
#2	Coronary Heart Disease [Title/Abstract]
#3	Coronary Diseases [Title/Abstract]
#4	Coronary Heart Diseases [Title/Abstract]
#5	Disease, Coronary [Title/Abstract]
#6	Disease, Coronary Heart [Title/Abstract]
#7	Diseases, Coronary [Title/Abstract]
#8	Diseases, Coronary Heart [Title/Abstract]
#9	Heart Disease, Coronary [Title/Abstract]
#10	Heart Diseases, Coronary [Title/Abstract]
#11	or/1–10
#12	Percutaneous Coronary Intervention [MeSH]
#13	Percutaneous Coronary Revascularization [Title/Abstract]
#14	Coronary Intervention, Percutaneous [Title/Abstract]
#15	Coronary Interventions, Percutaneous [Title/Abstract]
#16	Coronary Revascularization, Percutaneous [Title/Abstract]
#17	Coronary Revascularizations, Percutaneous [Title/Abstract]
#18	Intervention, Percutaneous Coronary [Title/Abstract]
#19	Interventions, Percutaneous Coronary [Title/Abstract]
#20	Percutaneous Coronary Interventions [Title/Abstract]
#21	Percutaneous Coronary Revascularizations [Title/Abstract]
#22	Revascularization, Percutaneous Coronary [Title/Abstract]
#23	Revascularizations, Percutaneous Coronary [Title/Abstract]
#24	or/12–23
#25	Coronary Restenosis [MeSH]
#26	Coronary Restenoses [Title/Abstract]
#27	Restenoses, Coronary [Title/Abstract]
#28	Restenosis, Coronary [Title/Abstract]
#29	or/25–28
#30	miRNA-21 [Title/Abstract]
#31	miR-21 [Title/Abstract]
#32	or/30–31
#33	diagnos^∗^ [Title/Abstract]
#34	Sensitivity [Title/Abstract]
#35	Specificity [Title/Abstract]
#36	ROC curve [Title/Abstract]
#37	or/33–36
#38	#11 and #24 and #29 and #32 and #37

### 2.4 Data collection and analysis

#### 2.4.1 Study selection

**Figure 1:**
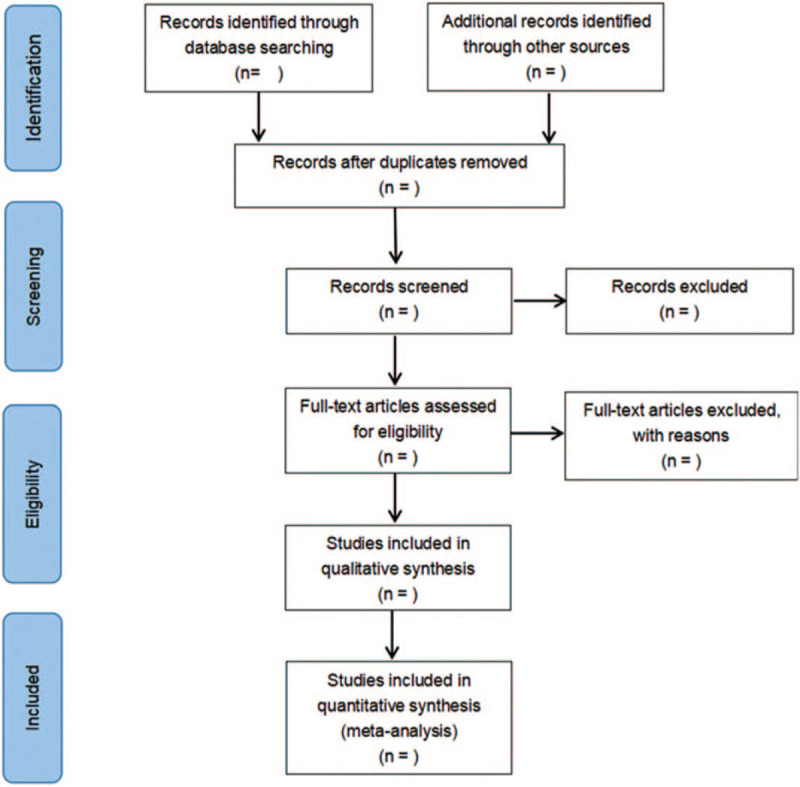
Flow diagram of literature retrieval.

Two researchers independently complete the literature screening, exclude the studies that obviously do not meet the inclusion criteria, and further read the abstracts and the full texts to determine whether they meet the inclusion criteria. The data included in the literature will be extracted and cross-checked. Disagreement should be solved by consulting a third researcher, thus reaching a consensus. The screening flow chart of this study is demonstrated in Figure 1.

#### 2.4.2 Data extraction

The data extraction form includes following items: first author, publication year, regions, sample size, sample types, control group, miRNA-21 detection methods, and data needed for diagnostic meta-analysis.

#### 2.4.3 Dealing with missing data

If the data of the required study are incomplete or not reported in the study, the researcher will contact the first author or other authors of the study by phone or email. If the required data are not available, we will perform descriptive analysis, instead of meta-analysis, and exclude these studies if necessary.

### 2.5 Quality assessment

The methodological quality of the included studies was assessed by following Quality Assessment of Diagnostic Accuracy Studies-2 criteria. Two reviewers independently and blindly checked the studies. Any discrepancies between the 2 reviewers were resolved by consensus.

### 2.6 Statistical analysis

All of the above statistical analyses were performed with Stata 16.0 (Stata Corp LLC, college station, TX, USA). We calculated the pooled SEN, SPE, Positive likelihood ratio, Negative likelihood ratio, Diagnosis odds ratio, and their 95% CI. In addition, the pooled diagnostic value of miRNA-21 through the summary receiver operating characteristic curve and Area under the curve was tested. The threshold effects were detected by using spearman correlation coefficient. The calculation of heterogeneity was caused by the non-threshold effect of Cochrane-Q and I^2^ values, and a fixed effect model (without obvious in homogeneity) or a random effects model (with significant heterogeneity) was employed to merge the data. The statistical test level was α = 0.05.

### 2.7 Subgroup analysis

In order to further investigate potential heterogeneity, subgroup analyses were conducted based on ethnicity, the source of miRNA-21 and sample size.

### 2.8 Sensitivity analysis

In order to test the stability of the meta-analysis results, we will adopt the one-by-one exclusion method to analyze the sensitivity of the results.

### 2.9 Reporting bias

The Deeks symmetry test was performed to detect whether there is a publication bias in the included studies.

### 2.10 Ethics and dissemination

Since the program does not include the recruitment of patients and the collection of personal information, it does not require the approval of the Ethics Committee.

## 3 Discussion

Stent intervention is easy to lead to vascular endothelial cell injury.^[26,27]^ This kind of damage causes the body to repair, while excessive repair may result in vascular restenosis.^[28]^ PCI treatment may do direct harm to the arterial wall, which may give rise to inflammatory reaction, thrombosis and intimal hyperplasia.^[29,30]^ These changes in vascular wall eventually bring neointimal formation and vascular remodeling, thus leading to restenosis after PCI. MiRNA has been widely used in cancer and other diseases, and has been the center of attention in the field of CHD.^[31–33]^ I has been proved that MiRNA-21 can promote cell proliferation and antagonize apoptosis, which affects the formation of neointima in vascular wall and participates in the pathological process of vascular stenosis.^[34]^ The researches of miRNA-21 on ischemic heart disease is still in its infancy, and the role of restenosis after PCI is still controversial. Although many studies indicated abnormal miRNA-21 expression in patients with coronary restenosis after PCI, a systematic review and meta-analysis will be warranted to compile and synthesize the available data, so as to address some of the questions

There are some limitations in this study. First of all, this study only searched the literature in Chinese and English, so there may be language bias. Second, most diagnostic tests were carried out in China and may be geographically biased. Third, different studies applied different diagnostic cut-off values, which may be the source of heterogeneity and may have an impact on the results. Fourth, the case-control design, rather than the cross-sectional design, was adopted in the literature included in this study. Therefore, both case population and control population do not come from the same specific population, thus resulting in bias.

The results of this review can provide clinical basis for the diagnosis of coronary restenosis after PCI in patients with CHD, and provide reference for further study on the possibility and development direction of coronary restenosis. With the rapid development of scientific research and the in-depth study of miRNA-21, it is believed that the application of this marker in the occurrence of coronary restenosis after PCI is worth looking forward to.

## Author contributions

**Data collection**: Xiaojun Ji and Haiyue Dai.

**Data curation:** Xiaojun Ji.

**Formal analysis:** Haiyue Dai.

**Funding acquisition:** Rui Zhou.

**Resources:** Jun Wang, Zhongping Shi.

**Software operating**: Haiyue Dai.

**Supervision:** Yiwei Huang.

**Writing – original draft:** Haiyue Dai, Rui Zhou.

**Writing – review & editing:** Haiyue Dai, Rui Zhou.
